# Educational—College Work

**Published:** 1896-10

**Authors:** 


### EDITORIAL.

### Educational—Co ge Work.

The time has again arrived when a great work is to be resumed, namely, the educational work, and this, too, all the way from the most elementary kindergartens to the highest university in the land—two classes are engaged, three, indeed—those who teach, those who are taught and the care-takers of both classes.

All classes of educational institutions are alive with energy, enthusiasm and industry in view of that which is before them. This may be said of those entering dental colleges as well as of any others. The numbers that have entered the dental schools during the last four years have been a surprise to all, teachers as well as non-teachers. During this time the aggregate number of students in our colleges has nearly doubled, and the indications are that the ratio of increase will be kept up this year, at least this may be inferred from the information that is current. An interesting inquiry : Why this rapid increase ? and how long is it to continue ?

In reply to these questions various considerations will enter : First, it is a fact that in none of the professions or pursuits of men has there been a more rapid gowth and development than in the art and science of dentistry, and this comes of a real necessity in the conditions of human society, that is being more and more largely recognized as the years go on : the value and importance of the teeth in the human economy were never so fully realized as at the present time, and with that recognition comes a desire for better things, and especially so when the efficiency of dental practice is so largely demonstrated as at the present day. Dentistry commands a respect to-day that was never accorded to it before, and on this account attracts the attention of the student more than ever.

Many who contemplate this as an occupation look on it as an easy one and yielding large pecuniary rewards, indeed, in some cases, expressions are indulged that indicate that this is the leading consideration: in entering the profession of dentistry it is well that this incentive be kept well in the background, lesc disappointment comes and stands as a persistent entity in the coming time.

The leading idea in taking up and following the liberal professions should be the accomplishment of the largest amount of good to humanity. The world needs that kind of help. Embarrassments and obstacles in other occupations, doubtless, have something to do in influencing young men to enter the dental colleges. And as crowds flock to the schools there is an incentive to organize more colleges, and competition is made the more aggressive and persistent.

To the second question: How long is this state of affairs to continue? Who knows? We will await the development of the future. We are not, however, in sympathy with the fear that the country is to be overrun with dentists.

Permit this suggestion: Let the determination of every teacher be, "I will do better and more thorough work than ever before;" and of every student, "I will make the most possible of myself and of my efforts during my college term, and prepare myself to bring honor to my alma mater, to my teachers and to my prospective profession, and to render the highest services to those who may, in the future, be placed in my care."

